# Lifestyle Factors and the Microbiome in Urolithiasis: A Narrative Review

**DOI:** 10.3390/nu17030465

**Published:** 2025-01-27

**Authors:** Antonios Koudonas, Stavros Tsiakaras, Vasileios Tzikoulis, Maria Papaioannou, Jean de la Rosette, Anastasios Anastasiadis, Georgios Dimitriadis

**Affiliations:** 1 First Department of Urology, School of Medicine, Faculty of Health Sciences, Aristotle University of Thessaloniki, 541 24 Thessaloniki, Greece; c3dw9@windowslive.com (A.K.); drstavros90@gmail.com (S.T.); bill1996tziko@gmail.com (V.T.); aanastaa@auth.gr (A.A.); gdimit@auth.gr (G.D.); 2 Laboratory of Biological Chemistry, School of Medicine, Faculty of Health Sciences, Aristotle University of Thessaloniki, 541 24 Thessaloniki, Greece; 3 Department of Urology, Istanbul Medipol Mega University Hospital, 34810 Istanbul, Turkey; jdelarosette@medipol.edu.tr

**Keywords:** urolithiasis, lithogenesis, prevention, lifestyle factors, microbiome

## Abstract

Urolithiasis represents one of the most common urologic diseases, and its incidence demonstrates, globally, an increasing trend. The application of preventive measures is an established strategy to reduce urolithiasis-related morbidity, and it is based mostly on the adaptation of lifestyle factors and pharmacotherapy. Furthermore, other research areas demonstrate promising results, such as the research on the microbiome. In the current review, we searched for the latest data on lifestyle–based prevention and microbiome alterations in urolithiasis patients. The majority of the proposed lifestyle measures are already included in the urological guidelines, while additional factors, such as vitamin D supplementation, seem to have a putative positive effect. From the microbiome studies, several microbial composition patterns and metabolic pathways demonstrated an inhibiting or promoting role in lithogenesis. Up to the present, stone prevention has not shown satisfying results, which suggests that lifestyle measures are not adequate. Moreover, microbiome studies are prone to bias, since microbes are strongly affected by numerous clinical factors, while the analysis procedures are not standardized yet. Analysis standardization and data pooling from extensive registration of clinical and microbiome data are essential steps in order to improve the existing prevention strategy with targeted microbiome manipulations.

## 1. Introduction

Urolithiasis comprises one of the most frequent urologic diagnoses worldwide, demonstrating a prevalence in the range from 1% to 13% in different geographical regions [1]. In general, urolithiasis-related incidence, disability-adjusted life years (DALYs), and deaths showed a substantial increase in the last decades, while the respective age-standardized rates decreased [1]. The increased urolithiasis incidence induces a continuously growing burden on healthcare systems globally.

The epidemiology of urolithiasis demonstrates a remarkable prevalence variation in terms of geography, which is, amongst other things, attributed to socioeconomic and climate factors [2]. Indeed, even in the same country, there are regions with remarkably higher urolithiasis incidence and prevalence, such as the southeastern states of the US [2]. An increased urolithiasis diagnosis is attributed to the adoption of better imaging through computer tomography (CT) since its systematic application has caused a detection bias by uncovering urolithiasis cases that would have gone undiagnosed in the past [3]. According to several researchers, the lack of a standardized stone classification system comprises an additional factor of deviation of the results of the available reports, rendering the assessment of urolithiasis trends more difficult [4].

Along with the geographical and temporal trends of urolithiasis, lithogenesis exhibits a significant tendency for male patients and a higher age at first diagnosis. However, this tendency seems to have been modified in the last few years. More precisely, the male-to-female ratio, which is reported in numerous studies, is 3:1, but in recent years, it seems to be narrowing since females demonstrate an increased rate of urinary stone-related symptoms, and, additionally a greater percentage of urinary stone-related mortality [5]. Moreover, the manifestation of urinary stone disease in younger patients is characterized by a continuously increasing frequency, while it exhibits an increasing trend even in the pediatric population, where from 1999 to 2008, an increase of 86% in emergency department visits was reported [6]. Interestingly, the stone composition has shown substantial modifications over recent years [7]. In particular, in female patients, the percentage of uric acid in urinary stones increased. Hence, the percentage of struvite, and additionally, the presence of apatite in calcium-containing stones of the same patient population diminished [7]. However, in male patients, the percentage of uric acid remained stable, while the presence of apatite in calcium-containing stones and the percentage of cystine and struvite stones increased [7]. The above modifications in stone disease epidemiology and stone composition suggest that urolithiasis cannot be completely attributed to crystallization processes, and several additional mechanisms are playing an active role in lithogenesis, yet, these mechanisms remain mostly unclear [8]. Since the above modifications of the stone disease have simultaneously taken place with changes in several aspects of daily routine during recent years, it is plausible to hypothesize that these aspects, such as lifestyle factors, may have metabolic implications, which are associated etiologically with lithogenesis. Furthermore, the microbiome is proposed as one of the novel models that can explain the accumulation of stone formation cases in specific patient categories [8].

Lifestyle factors include several modifiable elements of the environment/behavior of the population, such as dietary habits, physical activity, body weight balance, patterns of sleep, alcohol intake, and smoking. The above factors are gaining continuously increasing attention due to their association with several pathologic conditions, including various urologic diseases, on the rationale of applying a preventive strategy through their modification [9]. Urolithiasis has also been associated with the effect of specific lifestyle factors in numerous reports, so several urological associations already adopted the respective strategies in their guidelines. Indeed, the European Association of Urology (EAU) recommends several lifestyle modifications in the form of general preventive measures, many of which are supported by a high evidence level [10]. Similarly, the American Urological Association proposes several dietary recommendations with various evidence levels to prevent the recurrence of stone formation [11].

In contrast to lifestyle factors, the data on the association of the human microbiome with urolithiasis are more recent; therefore, there are no official preventive strategies relating to the manipulation of the microbiome. The microbiome is defined as the sum of symbiotic microbial cells, which are harbored mainly in the gut or other anatomic entities [12]. The role of the microbiome in health and disease has only recently emerged, based on the latest sequencing technologies, which allow a deeper understanding of microbiome composition and function [12]. The association of the microbiome with urological diseases was rendered more conceivable after the discovery of the urinary microbiome and the finding of microbes in the urine, which until a few years ago was considered sterile in healthy individuals [13]. In patients with urolithiasis, the microbiome is affected by several parameters, amongst others, lifestyle factors and diet [14]. These are also directly associated with lithogenesis, so the delineation of the net effect of microbiome alterations on stone formation is expected to be challenging.

The primary objective of the present literature review is to provide an update on the effect of lifestyle factors/the microbiome on urolithiasis. The secondary objective is to examine the perspective of applying microbiome manipulations for inhibiting the lithogenesis process.

## 2. Materials and Methods

**Figure 1:**
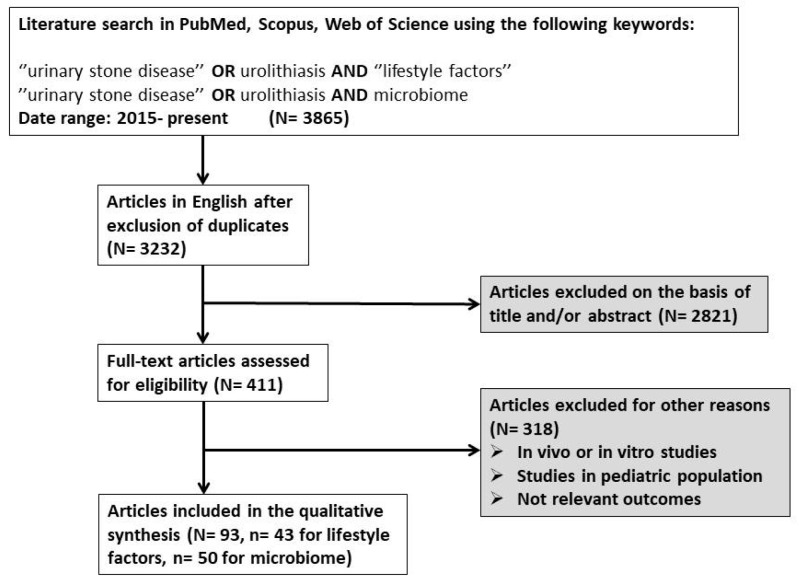
Flow diagram of the literature search.

In order to collect the relevant data for the current narrative review, we performed a literature search in three electronic databases (PubMed, Scopus, Web of Science) with the following search term combinations: (for lifestyle factors) = “urinary stone disease” OR urolithiasis AND lifestyle factors, (for microbiome) = “urinary stone disease” OR urolithiasis AND microbiome. Since the publication of data from next-generation sequencing relating to the microbiome and its association with urinary stone disease initiated in 2015, we included articles (original articles and meta-analyses) in the current review that were published from 2015 to the present, were written in English, and reported results only in adults (studies that reported only data from in vitro or in vivo methods, or from pediatric patients, were excluded). For the publications reporting on the effect of lifestyle factors on urolithiasis, we aimed to include reports focusing on the net effect of the above factors on lithogenesis. Therefore, we prioritized the studies that applied multivariate analyses or other methods of adjustment to exclude the effect of confounding factors. The above prioritization was not applicable for microbiome studies because of the very limited number of reports presenting adjusted results. After the exclusion of duplicates and irrelevant reports based on the title and/or abstract, we assessed 411 full-text articles, among which we excluded 318 articles since they were reporting results from laboratory experiments, animal testing, or clinical data from pediatric patients. Notably, several studies were excluded at the full-text assessment stage on the basis of reporting not relevant results for the scope of the current review (not representative of the general population, very small sample, etc.). Finally, we included 93 articles in the qualitative analysis (n = 43 for lifestyle factors, n = 50 for microbiome) (Figure 1).

## 3. Results

### 3.1. Lifestyle Factors

#### 3.1.1. Dietary Patterns

##### Dietary Intake of Oxalate and Calcium

The oversaturation of urine with minerals, such as calcium, oxalate (CaOx), and phosphate, either from the diet or internal metabolic processes, can result in the formation of urinary crystals. A substantial body of scientific evidence indicates that a significant number of kidney stones, particularly those composed of CaOx, primarily develop when CaOx crystals aggregate on Randall’s plaques or obstruct the Bellini ducts [15]. In 2020, Kumar et al. aimed to identify and measure the formation of oxalate nanocrystals after dietary oxalate ingestion by applying a high-resolution imaging technology, specifically the Nanoparticle Tracking Analysis (NTA) [16]. Their observations revealed a substantial increase in CaOx nanocrystals after the oxalate intake, suggesting that diets abundant in oxalate have the potential to induce nano crystalluria. In line with the above report, Siener et al. conducted a pilot study that aimed at evaluating the dietary effects on four patients diagnosed with primary hyperoxaluria (PH) [17]. Their findings led to the deduction that individuals with PH may benefit from limiting their intake of dietary oxalate and hydroxyproline. In 2016, Ferraro et al. conducted a research investigation to assess the potential relationship between vitamin C uptake and the risk of kidney stone formation [18]. Vitamin C, when ingested, undergoes conversion to oxalate and is subsequently excreted in the urine, a process with an established association with kidney stone development. The study incorporated data from the Nurses’ Health Studies (NHSs) I and II, involving 156,735 women, and the Health Professionals Follow-Up Study (HPFS), comprising 40,536 men. The results revealed that both total and supplemental vitamin C consumption was linked to a heightened risk of developing new kidney stones in men, while no such association was found in women.

Regarding the role of dietary calcium, Maric et al. investigated the calcium intake of 56 recurrent calcium stone formers compared to 78 healthy subjects [19]. The results showed that stone formers consumed less calcium-containing food products and, additionally, they had lower bone density than their healthy counterparts. The association of reduced calcium uptake with increased lithogenesis risk can be attributed to the binding of diet calcium with oxalate in the gut, which reduces the oxalate’s absorbability [15]. In conclusion, the restriction of dietary calcium may be harmful to both the lithogenesis tendency and the bone health of the patients.

##### Consumption of Animal-Based or Plant-Based Foods

Numerous prospective research investigations have explored the correlation between distinct dietary components and the susceptibility to nephrolithiasis. These inquiries have consistently reported an augmented risk linked to an increased intake of animal-derived protein. On the contrary, a reduced risk of kidney stone formation has consistently been observed in individuals who have a higher intake of fruits and vegetables. Dietary adaptations based on the above findings are stated as part of the general preventive measures against lithogenesis [10,11]. In 2016, Ferraro et al. conducted a prospective study examining the uptake of various protein types, potassium, and the animal protein-to-potassium ratio (a measure of net acid load) in relation to lithogenesis risk [20]. This research encompassed three distinct cohorts: the Health Professionals Follow-Up Study (HPFS) (42,919 participants), Nurses’ Health Study (NHS) I (60,128 individuals), and NHS II (90,629 participants). The study findings indicated that the risk of kidney stone formation is influenced by the specific sources of protein. Unlike proteins from vegetables and dairy, the consumption of non-dairy animal proteins was associated with a slightly elevated risk of kidney stones, especially in men and older women. These patient subgroups demonstrate an inherently higher lithogenesis risk on the basis of the hormone profile (promoting effect of testosterone; inhibiting effect of estrogens), which may be further increased by the diet pattern, as reported by another publication reporting on the same patient cohorts [21]. In summary, dietary patterns emphasizing higher fruit and vegetable consumption, or diets where these food groups outweigh animal protein, could be effective in reducing the risk of kidney stone formation. In a systematic review and meta-analysis conducted by Lin et al. in 2020, it was reported that meat consumption and animal protein were significant risk factors for kidney stones [22]. Conversely, applying a dietary regimen according to the Dietary Approaches to Stop Hypertension (DASH) model, which includes increased consumption of fruits, vegetables, and dairy while reducing saturated fats, was associated with a notable 31% improvement in the susceptibility to kidney stone formation. In 2019, a cross-sectional study involving 267 participants focused on the relationship between adherence to the Mediterranean diet (MedDiet) and urinary factors related to kidney stone development, specifically the risk of calcium salt and uric acid crystallization in urine [23]. The MedDiet includes mostly the consumption of olive oil, fruits, vegetables, nuts, fish, and legumes, while the consumption of meat, saturated fat, and sugar is limited. The researchers applied the Mediterranean Diet Score (MDS) to classify individuals into low (≤3), medium (4–5), or high (≥6) adherence to the MedDiet. Their results indicate that a higher degree of adherence to the MedDiet is associated with a reduced risk of calcium salt crystallization and uric acid crystallization in urine. This finding suggests that a dietary adaptation to the principles of the MedDiet may serve as a preventive measure or a means to reduce the occurrence and recurrence of kidney stones composed of calcium salts and uric acid. Similarly, Leone et al. observed a diminished risk of urolithiasis associated with adherence to the MedDiet [24]. They used a validated 136-item food frequency questionnaire to assess initial compliance with the Mediterranean dietary pattern. The study involved 16,094 participants over an average follow-up period of 9.6 years, with 735 new cases of nephrolithiasis identified during the above period. The risk of nephrolithiasis decreased with a higher consumption of dairy products and vegetables, while it increased with a higher ratio of monounsaturated fatty acids to saturated fatty acids. Increased adherence to the Mediterranean dietary pattern was linked to a reduced risk of developing nephrolithiasis. In 2017, a study by Ferraro et al. involved data from three extensive prospective cohorts and showed that adherence to low-risk dietary and lifestyle practices led to a reduction of more than 50% in the occurrence of kidney stones [25]. This study identified five adjustable risk factors (body mass index, fluid intake, DASH diet, dietary calcium, and intake of sugar-containing beverages) that seem to be associated etiologically with more than half of the newly developed kidney stones across the included cohorts. Consequently, adopting a diet rich in fruits, vegetables, and low-fat dairy products while ensuring an appropriate calcium intake was associated with a decreased risk of urolithiasis. In accordance with the previous report, a study conducted in Southern China involving 1519 patients unveiled an association between the consumption of pickled foods and animal proteins and the incidence of urolithiasis [26]. Conversely, the consumption of vegetables emerged as a significant and independent factor in the context of urinary stone prevention.

#### 3.1.2. Fluid Intake

##### Water Intake—Total Fluid Volume

Elevated liquid consumption has been widely recommended as a cost-effective and straightforward approach to prevent the formation of kidney stones. Both European and US guidelines for urolithiasis suggest adequate hydration to achieve a daily urine volume of ≥2.5 L [10,11]. Several studies specifically focus on water, whereas others investigate the role of various drinks primarily composed of substantial quantities of water. In 2016, a meta-analysis, which encompassed two randomized controlled trials (RCTs) involving 269 participants and seven observational studies involving 273,685 individuals, was conducted by Cheungpasitporn et al. [27]. It revealed that individuals with increased fluid intake exhibited a substantially reduced risk of developing kidney stones. In the RCTs, the pooled relative risk (RR) for kidney stone occurrence was 0.40 (95% CI 0.20–0.79), while in the observational studies, the RR was 0.49 (0.34–0.71). Moreover, the increased fluid intake was advantageous in terms of stone recurrence rates, since it diminished the respective risk compared to controls (RR = 0.4, 95% CI 0.2–0.79 and RR = 0.2, 95% CI 0.09–0.44 for RCTs and observational studies, respectively). In another meta-analysis, the investigators identified a substantial reduction in the likelihood of kidney stone development for every 500 mL increase in water intake [28]. This reduction was reflected in a RR of 0.93 (95% CI 0.87–0.98; *p* < 0.01). In 2017, Sagy et al. investigated the association of renal colic incidence with dietary adaptations during Ramadan, a period of fasting, which also includes abstinence from drinking, for Muslims during the ninth month of the Islamic calendar [29]. The analysis showed that one of every four renal colic events was attributed to Muslims, who represented 18.5% of the total population. Furthermore, new renal colic cases were associated significantly with the first two weeks of Ramadan fasting (RR= 1.27, 95% CI 1.03–1.5). More recently, a systematic review by Bao et al. evaluated the available reports on the effect of water intake as a preventive measure against stone disease and found no RCT on the above effect in the context of primary prevention [30]. One RCT assessed the role of water intake as secondary prevention, and the respective analysis showed that increased water intake may decrease stone recurrences and prolong the time to stone recurrence. The above results were characterized as low-certainty evidence by the authors.

##### Specific Liquids

As previously noted, when considering the potential measures for kidney stone prevention, several studies focused solely on water consumption, while others encompassed a wider array of drinks with substantial water content. The most common drinks investigated in terms of the effect on stone disease are coffee, tea, beverages, and alcohol. In a prospective cohort study conducted at the population level, researchers investigated the interplay between fluid intake, dietary components, and the risk of an individual’s first occurrence of kidney stones by analyzing data from hospital inpatient records [31]. The results showed that for every additional total fluid volume of 200 mL consumed per day, the likelihood of developing kidney stones diminished by 13%, as indicated by a hazard ratio (HR) of 0.87 (95% CI 0.85–0.89). Interestingly, the above protective effect was related to other fluids (tea, coffee, alcohol), but not to water intake.

Green tea is popularly consumed for its presumed health advantages, mainly due to its rich concentration of antioxidant polyphenols. However, it is essential to recognize that tea also contains a substantial amount of oxalate. In a prospective study involving 12 healthy male participants, the researchers evaluated the influence of green tea on urine oxalate [32]. According to the respective protocol, the participants consumed green tea for seven consecutive days, and their 24-h urine chemistry was compared to the urine chemistry results under normal dietary conditions. The analysis showed a statistically significant rise in oxalate excretion in urine, providing evidence that green tea intake was associated with increased urine oxalate. Similarly, Rode et al. evaluated the differences in the 24-h urine chemistry of 273 hypercalciuric stone formers, who consumed green tea to various extents [33]. The analysis demonstrated no differences in the urine constituents, which are considered stone risk factors, between green tea drinkers and non-drinkers. Notably, COM stones were significantly decreased in female green tea drinkers compared to non-drinkers, while no stone composition difference was found in males. A retrospective study by Chen et al. investigated the connection between tea consumption and the prevalence of renal stone disease [34]. The results revealed that a daily tea intake of ≥ 240 mL is associated with a reduced risk of developing kidney stones (OR = 0.84, CI 0.71–0.99, *p* = 0.037). Additionally, when considering both the quantity and duration of tea consumption, a total intake of ≥ 20 cup-years is linked to a decreased risk (OR = 0.79, CI 0.66–0.94, *p* = 0.008) for renal stone disease. These findings align with another study, which was conducted in a large prospective Chinese cohort and documented that the consumption of green tea is linked to a reduced risk of developing new kidney stones [35]. Importantly, the above effect was analogous to the tea consumption extent (dose) and appeared to be more pronounced among male participants. On the contrary, Haghighatdoost et al. compared the tea consumption of patients with CaOx stones and healthy controls and found that individuals who consumed daily four or more glasses of black tea had an elevated risk of developing CaOx stones when compared to those who consumed fewer than two glasses of tea per day (OR = 2.73; 95% CI: 1.50–4.99) [36].

Notably, the tea consumption method (addition of milk, sugar, etc.) varied significantly across the respective studies, which may explain the different absorbability of the lithogenesis-relevant tea constituents [37]. Moreover, different tea preparations demonstrate substantial differences in the amount of oxalate, which depends on various factors, such as the origin, quality, process (black tea has the highest oxalate content), and time of tea harvesting [37]. The above oxalate concentration differences combined with the genetic polymorphism relating to oxalate metabolism render the methodology of the respective reports suboptimally standardized and pose limitations on the delineation of the effect of tea consumption on lithogenesis risk. Additionally, the discrepancies regarding the effect of tea consumption have been observed analogously in other studies on various diseases, such as the association of tea consumption with hyperuricemia [38].

##### Alcohol

Alcohol consumption has an unclear relationship with kidney stone formation since it induces several negative effects in multiple organs, and, at the same time, its diuretic effect could decrease the concentration of stone formation-related urine constituents. In 2015, Wang et al. performed a comprehensive systematic review and meta-analysis, which included eight observational studies, and documented a significant association between alcohol consumption and a reduced risk of urolithiasis (OR = 0.683, 95% CI 0.577–0.808) [39]. Furthermore, in the dose–response analysis, urolithiasis risk displayed a 10% reduction for each additional daily consumption of 10 grams of alcohol (OR = 0.898, 95% CI 0.851–0.948).

#### 3.1.3. Metabolism and Body Homeostasis

##### Factors Affecting Calcium Metabolism

CaOx and calcium phosphate are the most common types of kidney stones; thus, any factor affecting calcium metabolism may play a role in stone formation. Vitamin D is strongly involved in calcium (as well as phosphorus) metabolism by promoting its intestinal absorption; thus, it is able to increase urinary calcium [40]. There are two vitamin D sources: sun exposure and diet. Increased vitamin D intake has been associated with a reduced risk of fractures and improved muscle function, among other effects, so supplementation has even been proposed as a public health measure [40].

In 2016, Ticinesi et al. investigated the association between vitamin D deficiency (<20 ng/mL) and idiopathic calcium nephrolithiasis (ICN) [41]. A total of 884 patients with ICN and 967 controls were selected. The prevalence of vitamin D deficiency (<20 ng/mL) was 56% in stone formers (SFs) and 44% in controls, with median levels of 18 ng/mL vs. 23 ng/mL. There was a statistically significant association between vitamin D deficiency and odds of nephrolithiasis (OR = 2.29), while 25-OH-D levels were not different in hypercalciuric and normocalciuric stone formers (18 vs. 19 ng/mL). Vitamin D levels were significantly affected by the timing of blood sample collection. Serum parathormone (PTH) proved to be inversely correlated with 25-OH-D, whereas serum ionized calcium was significantly higher in SFs with 25-OH-D ≥ 30 ng/mL than in SF with a vitamin D deficiency (<20 ng/mL). Urinary calcium excretion was, however, unaffected by vitamin D status. Stone composition assessed by infrared spectrophotometry was available in 325 patients. CaOx was the most common type (67%), followed by mixed calcium oxalate/phosphate (28%) and calcium phosphate alone (5%). As a conclusion, the authors found that vitamin D deficiency was independently associated with an increased risk of ICN.

In the same year, Johri et al. studied the prevalence of vitamin D deficiency and the effect of cholecalciferol (vitD3) supplementation on stone risk in a cohort of idiopathic stone formers (ISFs), by comparing vitD-deficient (≤12 ng/mL) with vitD-insufficient (13–30 ng/mL) or vitD-replete (>30 ng/mL) patients and by investigating the effect of giving vitamin D3, 20,000 IUs, orally, weekly for 4 months, to 37 vitD-deficient subjects [42]. The cohort consisted of 456 subjects, and among them, 31% were vitD-deficient (142/456), 57% were (259/456) insufficient, and 12% were replete (55/456). Baseline 24 h urinary parameters, which were related to stone risk and were expressed as concentration ratios over urine creatinine (such as calcium, oxalate, citrate, uric acid, and phosphate), were measured. The aforementioned vitamin D3 administration was associated with an increase in both urinary calcium and phosphate, as well as a decrease in serum PTH levels. Thus, a reasonable strategy includes monitoring urinary calcium excretion in vitamin D-supplemented stone formers, because it may reveal underlying hypercalciuria in some treated patients. In another study, Malihi et al. analyzed the side effects, which were related to calcium metabolism, specifically hypercalcemia (>10.2–11 mg/dL, depending on the target population), hypercalciuria (urinary calcium-to-creatinine ratio > 0.3 or a 24 h urinary calcium excretion > 250 mg/d for women and >275–300 mg/d for men), and kidney stones, in adults who were given vitamin D supplements for ≥ 24 weeks compared with subjects in the placebo arm [43]. In a total of 48 studies with 19,833 participants, long-term vitamin D supplementation resulted in increased risks of hypercalcemia (RR = 1.54) and hypercalciuria (RR = 1.64), which were not dose-related. However, it did not increase the risk of kidney stones (RR = 0.66). The effect of vitamin D supplementation on the risk of hypercalcemia, hypercalciuria, or kidney stones was not modified by baseline 25-hydroxyvitamin D, vitamin D dose and duration, or calcium co-supplementation.

In 2017, Ferraro et al. prospectively studied the association between the intake of vitamin D and the risk of incident kidney stones by analyzing a total of 193,551 participants of the HPFS and NHSs I and II cohorts [44]. The included individuals were divided into categories of total and supplemental vitamin D intake. During a follow-up of 3,316,846 person-years, there were 6576 incident kidney stone events. A suggestion of higher risk in NHS II was observed only in the category with the highest intake of vitamin D3 (≥1000 IU/d). Thus, vitamin D intake in standard doses was not statistically associated with the risk of kidney stone formation, though a higher risk with higher doses than those studied here cannot be excluded. In 2019, Malihi et al. investigated the incidence of kidney stones and hypercalcemia events in a large, population-based (5110 participants), double-blind RCT of monthly vitamin D supplementation with 100,000 IUs [45]. During a median follow-up of 3.3 years, 158 participants, aged 50–84, reported a kidney stone event (76 from the vitamin D arm, 82 from the placebo arm) (HR = 0.90), with 18 urolithiasis events in the hospitalization records (7 from the vitamin D arm, 11 from the placebo arm) (HR = 0.62). From the subsample’s (8.5% of the initially recruited participants) annual blood test, there was no case of hypercalcemia in the vitamin D arm compared with one in the placebo arm. So, monthly supplementation with 100,000 IUs of vitamin D3 did not affect the incidence rate of kidney stone events or hypercalcemia. Although the existing evidence demonstrates that vitamin D supplementation as a measure against vitamin D deficiency seems to be beneficial in terms of lithogenesis risk, the differences in adaptation patterns against vitamin D scarcity must be taken into consideration, since various human populations utilize the available vitamin D differently, which can render supplementation unnecessary or harmful [46]. More precisely, the evolutionary adaptation of human populations with different geographies to various degrees of vitamin D availability renders the indication for supplementation less clear. According to a report by Carlber et al., the dispute about the optimal vitamin D status can be explained by the concept of an individualized vitamin D response index, which characterizes the degree of the molecular response to vitamin D supplementation [47]. Consequently, high responders may be exposed to the risk of hypercalciuria and lithogenesis under supplementation with standard vitamin D doses. Similarly, contemporary guidelines for vitamin D supplementation must take into consideration various factors, such as age group, ethnicity, and latitude of residence [48].

##### Role of Metabolic Disorders

Body composition and the body mass index (BMI) reflect the status of metabolism balance, and since lithogenesis seems to be triggered by metabolic disorders, there is a frequent coexistence of body composition abnormalities with urinary stone disease. The above association is also included in the general preventive measures in the form of recommendations for retaining a normal BMI [10]. In 2014, Shavit et al. investigated urinary metabolic parameters, stone composition, and stone formation risk in overweight (OW) kidney stone formers (KSFs) when compared with normal-weight and obese KSFs [49]. Patients were divided into three categories based on their BMI (<25, 25–30, and >30). A total of 2132 patients were included. There were significantly higher levels of urinary calcium, oxalate, citrate, uric acid, and sodium, and lower urinary pH in OW and obese KSFs. Stone composition data (n = 640) revealed a significantly higher incidence of uric acid stones among OW and obese groups, while a higher probability of stone formation of CaOx, uric acid, and such mixed stone types was detected in these groups. Thus, appropriate evaluation and follow-up may be justified not only in obese but also in OW patients who are at risk of increased stone formation.

In 2015, Akarken et al. examined the relationship between stone disease and the amount of visceral adipose tissue measured with unenhanced CT [50]. In total, 149 stone formers were analyzed for age, gender, BMI, amount of visceral and subcutaneous adipose tissue, and serum levels of low-density lipoprotein and triglyceride and were compared to 139 healthy individuals. Hypertension, hyperlipidemia, obesity (BMI > 30), and the ratio of visceral fat tissue to subcutaneous fat tissue were identified as factors increasing the risk of kidney stone formation. Visceral abdominal area (VAA > 180 cm^2^) has emerged as a new independent risk factor for urolithiasis. Hyperlipidemia, in particular, was detected in more than 30% of kidney stone patients, with significantly higher lipid levels in this group compared to the patients without kidney stones. In 2016, Yoshimura et al. evaluated BMI and the incidence of kidney stones in Japanese men [51]. A total of 5984 males aged 20–40 years old underwent a baseline evaluation, and 4074 of them, who were free of kidney stones at baseline, underwent a second medical evaluation. Their BMI was calculated from their measured height and weight, and men were categorized into tertiles. After an average follow-up duration of 19 years, 258 participants developed kidney stones. Using the lowest BMI group (15.9–21.6) as a reference, the HRs for the second (21.7–23.7) and third BMI tertiles (23.8–35.6) were 1.28 and 1.41, respectively, suggesting a positive correlation between BMI and kidney stone risk. The potential mechanism lies in insulin resistance, which suppresses calcium reabsorption by acting on renal tubules and promotes calcium excretion, plus an increase in urinary oxalate and a decrease in urinary citrate along with higher levels of urinary pH through a disturbance in ammonia formation and Na/H activities.

In 2018, Aune et al. attempted to clarify the association between adiposity, diabetes, physical activity, and the risk of kidney stones through a systematic review and meta-analysis of 13 cohort studies [52]. The results were expressed as RRs. Positive associations between adiposity (summary RR of 1.21 per 5-unit increments in BMI and 1.16 per 10 cm increase in waist circumference, 1.06 per 5 kg increase in body weight, and 1.12 per 5 kg of weight gain), diabetes (summary RR was 1.16), and the risk of kidney stones was found, but no association with physical activity (summary RR of 0.93 for high vs. low) was confirmed. In 2019, Chao et al. investigated whether frailty influences the probability of patients with diabetes mellitus (DM) to develop urolithiasis [53]. By using a modified FRAIL scale (fatigue, resistance, ambulation, illness, loss of body weight), they examined the relationships between frailty, its severity, and the risk of urolithiasis among diabetic patients. From a total of 525.638 patients, 64.4% were not frail, while 28.5%, 6.6%, and 0.6% had 1, 2, and ≥ 3 FRAIL items at baseline. After 4.2 years of follow-up, 3.4% (18.034) experienced incident urolithiasis. Patients with DM having at least one FRAIL criterion exhibited a significantly higher urolithiasis risk compared with non-frail patients, with the probability of stone formation increasing stepwise with the severity of frailty. Illness items emerged as a significant risk predictor. The most plausible reasons were resorptive hypercalciuria (due to lower physical activity and immobilization), higher urinary calcium excretion (increased protein intake leads to acid production plus enhanced intestinal calcium absorption), and dehydration (osmotic diuresis, reduced fluid intake). Thus, the authors concluded that treating frailty may potentially reduce their risk for urolithiasis.

In 2021, Iwasa et al. observed an increase both in patients with urinary stones and in the prevalence of metabolic syndrome, so they tried to determine the prevalence of markers of metabolic disorders in patients with urinary tract stones [54]. In a retrospective study of 715 patients, obesity (BMI > 30) and sarcopenia (age-related reduction in skeletal musculature) rates were higher in patients undergoing surgery for urinary stones than in the general population. Metabolic syndrome has been linked with the Western diet, which causes an acid overload and leads to hypertension, dyslipidemia, and hyperglycemia, posing a known risk factor for urinary tract stones, while sarcopenia has a high risk of osteoporosis, which increases urinary calcium excretion. The prevalence of these two factors was distributed differently in different age groups (higher rate of obesity in the middle-aged group, 40–60 years old, and higher rates of sarcopenia in the elderly group, in their 80s). Based on these findings, the authors recommended that individuals should apply a balanced diet routine and keep up core muscle training, as well as not reduce protein intake as they age. In 2022, Crivelli et al. assessed the extent to which obesity and neighborhood characteristics jointly contribute to urinary risk factors for kidney stone disease by retrospectively evaluating 24 h urinary measurements and obtaining socioeconomic status (SES) data for a total of 1264 kidney stone patients [55]. Obesity was associated with increased odds of multiple stone risk factors (OR = 1.61) (urine volume, calcium, oxalate, citrate, pH, and uric acid) and multiple dietary factors (OR = 1.33) (urine sodium, potassium, magnesium, phosphorus, ammonium, sulfate, and urea nitrogen). However, these interactions did not vary significantly by SES/family structure or housing characteristics. Notably, a significant inverse correlation between SES and 24 h urine sodium was demonstrated in the study.

In 2022, Siener et al. evaluated the effect of a conventional, low-energy-containing diet (1200 kcal, 15–20% protein intake, 30% fat intake, 50–55% carbohydrate intake) with (MR group applied replacement of two or three meals per day with meal products in the form of shakes, soups, or bars) or without (C group) meal replacement on risk factors for stone formation in a RCT of 78 OW women without a history of urolithiasis [56]. Anthropometric, clinical, biochemical, and 24 h urinary parameters were collected at baseline and after 12 weeks. The relative supersaturation of CaOx decreased significantly in both groups (due to increased urine volume), but a significant decline in serum uric acid concentration and relative supersaturation of uric acid was observed only in the MR group (along with higher relative weight loss and improved cardiometabolic risk). Increased urine volume plus a decline in urine density and a mild, non-significant increase in urinary pH may explain the decreased relative supersaturation of uric acid. Finally, the low-energy-containing diet with meal replacement showed significant advantages over the low-energy-containing diet alone.

#### 3.1.4. Environmental and Behavioral Factors

##### Role of Behavioral Patterns

Healthy behavioral patterns have also been associated with the prevention of urinary stone disease, so respective behavioral adaptations are also recommended as a form of general prevention against stone disease occurrence/recurrence [10]. In 2015, Soueidan et al. explored possible relationships between selected lifestyle factors and recent (<6 months) symptomatic urolithiasis (RSU) by administering questionnaires regarding socio-demographics, medical history, physical activity, diet, and smoking status in 163 stone clinic patients [57]. A total of 57 patients (35%) reported RSU, while 52 participants met the sum of the physical activity guidelines (15, 14, and 15 patients met the criteria for walking and moderate or vigorous activity, respectively). Participants with RSU had higher rates of smoking (7% vs. 21%, *p* = 0.02) and had 3.8 times the odds of being current smokers (8.5 times after controlling for age and other confounders), so current smoking was a potent predictor of RSU. Thus, the authors recommended that, when desired, smokers should be referred for smoking cessation. Two mechanisms have been proposed. Smoking induces increased secretion of plasma anti-diuretic hormone, which decreases the urinary volume and promotes the urinary supersaturation of crystals. Secondly, increased oxidative stress and renal injury are inflicted by reactive oxygen species production. Physical inactivity was not associated with RSU status. No differences in terms of BMI, added salt and water intake, oxalate, and protein consumption between groups were recorded. In the same year, Ferraro et al. performed a data analysis from three large prospective cohorts (HPFS, NHS I, NHS II) and assessed the HRs of incident stones among participants within different categories of physical activity (PA) and energy intake (EI), after adjustment for calcium supplement use and other confounders [58]. From a total of 215,133 participants and up to 20 years of follow-up, 5355 incident cases of kidney stones, predominantly CaOx stones, were recorded. After multivariate adjustment, there was no significant association between either PA or EI and incident kidney stones.

In 2017, Hess et al. evaluated two groups of recurrent stone formers (RCSFs) in terms of understanding the stone formation mechanism and adherence to therapeutic recommendations (diet, lifestyle, drug treatment) at least 3 months after a 60–75 min consultation to explain metabolic evaluation and therapeutic measures [59]. The main general recommendations, which were given to RCSFs, were increased fluid intake (at least 2 L of urine per day), avoidance of excess oxalate intake in combination with calcium intake, 1200 mg calcium intake per day, restriction of animal protein to one serving per day or 1 g per kg body weight, at least three servings of alkali-rich products, such as fruits and vegetables, stress reduction, and alkali therapy if hypocitraturia or distal renal tubular acidosis was present. Results revealed that both groups performed similarly, and the pathophysiologic explanations of stone disease were understood to the extent of >80% by 2/3 of RCSFs. After 3 months, perfect adherence to the recommended treatment was more frequent on alkali citrate (3.3 times more prevalent) than on dietary/lifestyle interventions. Increasing calcium and fluid intake were the most popular dietary measures, whereas reducing psychosocial stress was the least popular one. In the same year, Fabregas et al. conducted a retrospective study to investigate the possible association between chronic stress and stone recurrence [60]. In the above study, the researchers recruited 128 participants, who were assessed for chronic stress levels as well as for stress caused by their stone episodes per se, and they calculated urinary ratios and quotients that are regarded as diagnostic indicators of stone risk (including Ca/Cr, Ox/Cr, Mg/Cr, Cit/Cr, urate/Cr, and citrate/magnesium/calcium ratios), the activity product quotient for calcium oxalate (CaOx), and the relative supersaturation of CaOx, brushite, and uric acid. Although recurrent stone formers (RCSFs) had more stressful life events, with a greater intensity of perception, than first-time stone formers (FSs), there were no significant differences between the groups regarding any of the urinary risk factors. Consequently, a direct causal link between stone recurrence and stress was not demonstrated. An inherent problem in attempting to solve the stress–stones dichotomy, though, is the need to disentangle alterations in risk factors that arise from lifestyle stress and those arising from stone episodes themselves.

##### Role of Climatic, Occupational, and Social Factors

Regarding climatic factors and based on the fact that the incidence of symptomatic stone events reaches its peak in summer (due to concentrated and acidified urine that promotes supersaturation and nucleation), many researchers aimed to investigate further the association of climatic parameters with stone formation. According to a study published by Park et al. in 2015, a seasonal trend characterized the incidence of symptomatic stone disease, which demonstrated a peak plateau between July and September, and a rapid decline after September [61]. After adjustment for confounding factors, ambient temperature was the only climatic parameter significantly affecting the risk for symptomatic stone disease. The analysis demonstrated that by considering 18.4 °C as the threshold temperature, every ambient temperature increase by 1 °C augmented the risk for a symptomatic stone event by 1.71%. In 2016, Choi et al. evaluated the different climatic factors in urban and rural areas that may affect the incidence of urolithiasis [62]. Data (gender, age, date of diagnosis, geographic region, and daily weather data) between 2009 and 2013 were grouped by population density. The primary outcome was the incidence rate in each region. The secondary outcomes were differences between groups and RRs of climatic factors, while the tertiary outcome was RRs of urolithiasis presentation cumulated over a 20-day lag period associated with the mean daily temperature. The incidence rates of urolithiasis tended to increase annually in most regions, with a total number of 1,452,671 urolithiasis events, whereas the urban group showed a higher mean temperature, lower amount of rainfall, higher wind speed, and lower mean relative humidity than the rural group, with RRs that increased gradually with increasing temperature. These results revealed that regional differences in climatic factors, especially temperature, may provoke a gap in urolithiasis events between the urban and rural areas. This implies that urbanization may affect the prevalence of urolithiasis and that global warming can also increase urolithiasis events. Nevertheless, the key connection between urban areas and urolithiasis is not clarified adequately from the above results. Beyond climatic factors, individuals living in urban areas differ in additional parameters compared to rural areas, such as dietary patterns. According to a report by Yang et al., the differences in dietary habits between urban and rural populations are significant and associated with adverse conditions, such as obesity and hypertension [63]. Provided that the above conditions are independently correlated with lithogenesis, the increased urolithiasis incidence in urban areas may be attributed to factors additional to the climatic parameters.

In 2017, Guo et al. performed a meta-analysis to determine whether cadmium exposure is associated with urolithiasis in humans [64]. A total of 88,045 participants were identified (six observational studies, occupational exposure n = 4, and dietary exposure n = 2) and were stratified into two categories according to cadmium assessment results. The risk of urolithiasis increased significantly, by 1.32 times, at higher cadmium exposure (OR = 1.32), and this association was higher with occupational exposure (OR = 1.56). Meanwhile, no association was observed between cadmium exposure and urolithiasis risk with dietary exposure. Cadmium is a widespread pollutant with known toxic effects, such as fractures and prostate cancer. It has various sources, including battery manufacturing, metal processing industries, contaminated soils, and foods like bread, cereal, and vegetables. The potential mechanism is increased bone resorption and urinary calcium excretion or a direct toxic effect on renal tubular cells, leading to hypercalciuria. The summary of the study stated that the government should accelerate the sanitization of contaminated soils and reduce exposure to pollutants. In the same year, Lotan et al. conducted an assessment of hydration status within a group at elevated risk, consisting of steel manufacturing plant employees, who are exposed to a hot work environment [65]. This evaluation involved the examination of end-of-shift urine osmolality (referred to as "post-shift") and the analysis of 24 h urinary parameters known to be associated with the risk of kidney stone formation. The study revealed a notably heightened prevalence of kidney stone disease among this particular subset of steelworkers when compared to the documented occurrence of kidney stones in the general population. Regarding the effect of the social environment, Bayne et al. hypothesized that socioeconomic parameters impact kidney stone severity at intake to referral centers and attempted to determine social factors associated with advanced stone disease (defined as unilateral stone burden > 2 cm) at the time of presentation to a regional stone referral center [66]. A retrospective review of the prospectively collected data on patient age, gender, BMI, diabetes, race, language, education level, infection, distance, income, referring regional urologist density, American Society of Anesthesiologists score, and stone analysis was performed. A total of 1142 patients were included in the study, including 197 with a unilateral stone burden >2 cm. Obesity, lower educational level, increased distance from the referral center, and symptoms of infection predicted for advanced stone disease. Moreover, among 191 patients with available stone analysis data, stone type (non-CaOx stones), income, and urologist density in their geographic regions were significantly associated with stone burdens > 2 cm.

### 3.2. Microbiome

#### 3.2.1. Gut Microbiome

##### Studies Referring to the Oxalate-Degrading Pathway

**Figure 2:**
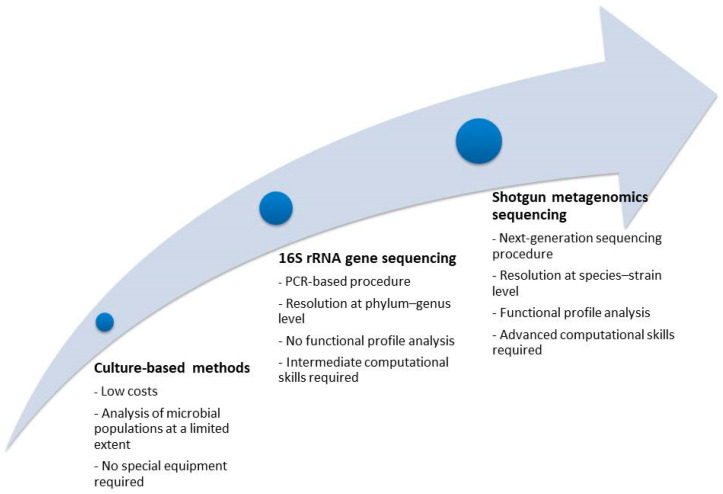
The evolution of microbiome research.

Oxalate comprises a basic component of the most common stone composition (CaOx), and its source can be its endogenous production, or the intestinal absorption from oxalate-containing foods [10]. The increased oxalate concentration in the urine of patients with increased oxalate absorption is known as enteric hyperoxaluria and is dependent on several factors, such as the functionality of the gastrointestinal tract (GI), the presence of inflammatory diseases of the GI, and any previous intestinal resection [10]. Interestingly, an important factor in the management of enteric oxalate is the gut microbiome. The evolution of microbiome research (Figure 2) has allowed the delineation of microbes with oxalate-degrading activity, and an increasing number of studies are reporting on the association of enteric microbes with oxalate metabolism. In 2016, Suryavanshi et al. analyzed the microbiome characteristics in fecal samples of 24 stone formers compared to 15 healthy subjects by DNA extraction and 16S rRNA gene sequencing [67]. Specific bacterial classes/phyla were found to be enriched in the patient group (*Firmicutes*, *Proteobacteria*), while others were decreased (*Bacteroidetes*, *Cyanobacteria*). Moreover, the enzymes involved in oxalate degradation were increased in the patient group, while *Oxalobacter formigenes*, which is well known for its energy dependence on oxalate, was found in both comparison groups without any significant difference in abundance. Finally, the relative contribution of *Oxalobacter formigenes* in oxalate degradation was found to be reduced, while other oxalate-metabolizing microbes were enriched in the patient group. In 2017, Ticinesi et al. compared the results of gut microbiome analysis of 55 recurrent calcium stone formers to 53 healthy controls with similar clinical characteristics [68]. The fecal microbiome of stone formers showed reduced biodiversity and a lower representation of genes involved in the metabolism of oxalate. The metagenomics sequencing revealed that the oxalate-related metabolic processes were referring to microbial taxa, which were never associated with oxalate-degrading activity in the past, and the respective genes were underrepresented in stone formers. Three microbial taxa (*Faecalibacterium*, *Enterobacter*, *Dorea*) were significantly reduced in the gut of stone formers, while *Oxalobacter* showed no difference between the comparison groups. The findings of the above study were in line with the conclusions of a meta-analysis by Batagello et al. [69], where the pooling of data showed that *Oxalobacter formigenes* colonization cannot predict stone disease risk or oxalate excretion in urine. Moreover, it seems that a broader diversity of bacteria is associated with oxalate metabolism, such as the taxa *Bacteroides*, *Ruminococcus*, *Bifidobacterium*, *Coprococcus*, *Lactobacillus*, *Oscilospira,* and *Parabacteroides*, which were detected in healthy individuals. This fact explains the unclear results of probiotics prescription, which reinforces only specific species of oxalate-degrading bacteria in the gut. The finding of reduced microbe diversity, a state which is recognized as a sign of dysbiosis and can be attributed to the multiplication of several dominant species, was again reported by Choy et al. after comparing the bacterial populations of the fecal samples of 17 recurrent formers to 17 healthy subjects [70]. In contrast to other reports, the stone formers were characterized by a lower abundance of *Oxalobacter formigenes* and reduced representation of genes associated with oxalate metabolism, while the comparison of other known oxalate-degrading bacteria did not show any significant differences in terms of abundance. In 2018, Suryavanshi et al. reported the results of comparing the populations of other-domain microbes and the standard functional eubacteria of the gut [71]. Eleven members of archaea, seven of eukarya, and six fungal species were found to be abundant in the control group, while they were absent among the oxalate stone formers. Regarding the eubacteria, the oxalate-degrading microbes were enriched in the patient group, but the well-known oxalate metabolizers (*Oxalobacter formigenes*, *Lactobacillus plantarum*) were reduced in the same group. The genes related to oxalate metabolism were increased in the patient group. In 2019, Miller et al. analyzed the microbial population of 17 stone formers and 17 healthy subjects with comparable clinical characteristics [72]. At the phylum level, a significant reduction in *Tenericutes* was found in the gut of the patients, while the differences between comparison groups in terms of gut microbiota composition were additionally sex-dependent. Regarding the oxalate-metabolizing microbes, specific taxa (*Ruminococcus*, *Oscillospira*) showed a selective presence in healthy individuals only, while other taxa showed no differences between comparison groups. More recently, Tavasoli et al. compared three groups (30 recurrent stone formers with CaOx stones and hyperoxaluria, 30 recurrent stone formers of the same composition without hyperoxaluria, and 30 healthy individuals) to uncover the association of *Oxalobacter formigenes*, *Lactobacillum,* and *Bifidobacterium* in the gut microbiome with hyperoxaluria and stone formation [73]. The results showed a significant negative association of the relative abundance of *Oxalobacter* only with the presence of stones, while the presence of the two other microbes did not correlate to hyperoxaluria or stone formation. In 2020, Ravikumar et al. tested the hypothesis of the etiological association of the absence of *Oxalobacter formigenes* with stone formation and reduced bone density in stone formers on the rationale of impaired calcium absorption after the formation of non-absorbable CaOx complexes in the gut [74]. Indeed, more healthy subjects were colonized with the above microbe and had an increased bone density. The researchers of the study did not apply a direct recognition method of *Oxalobacter* as in the majority of the recent reports. According to a report by Chen et al. [75], oxalate-degrading microbes may be suppressed by specific foods, which can explain the increased tendency for stone formation among the respective patients. In the above study, five microbial genera were identified as biomarkers for CaOx stones. Among them, the abundance of the protective genera (*Akkermansia*, *Lactobacillus*) was negatively correlated with the increased consumption of tea, suggesting an alternative mechanism of the association of tea consumption with lithogenesis. In 2023, Liu et al. analyzed bioinformatical data, which included 16S rRNA sequencing results from fecal samples, to investigate the eventual causal associations between specific microbial populations and stone disease [76]. The above analysis did not demonstrate any correlation between the genus *Oxalobacter* and lithogenesis. On the contrary, several other microbial classes were found to exert either a triggering effect for stone formation (genus *Haemophilus*, genus *Ruminococcaceae*, genus *Subdoligranulum*), or to have an inhibiting role against stone disease (order *Actinomycetales*, family *Actinomycetaceae*, family *Clostridiaceae*, genus *Clostridiumsensustricto,* and genus *Hungatella*). No reverse effect from the stone disease to the gut microbiome was found.

##### Studies Referring to Other Alterations of the Gut Microbiome

Several studies, which were found during our literature search, reported results unrelated to oxalate metabolism, or related to other metabolic shifts with a putative role in stone formation. In 2016, Stern et al. enrolled 23 stone formers and 6 healthy individuals in a study protocol to uncover the alteration that characterizes the gut microbiome of the patient group [77]. The relative abundance of the two species was found to be significantly different; namely, *Prevotella* was 2.8 times more abundant in the control group, and *Bacteroides* was 3.4 times more abundant in the patient group. The abundance of the above species retained a statistically significant association with stone formation after adjustment for age, gender, and other clinical parameters. The comparison of bacterial abundance between uric acid and non-uric acid stone formers showed no significant difference. In 2017, Tang et al. compared the microbial populations of 13 stone formers and 13 healthy subjects, which were matched in terms of age and race [78]. The number of the observed microbe species in the patient group was lower, while the composition of the species between comparison groups was significantly different. More precisely, 20 microbial genera were found in different abundances, and many of the genera that were found to be enriched in the patient group have a proinflammatory role (*Megamonas*, *Phascolarctobacterium*, *Sutterela*). Moreover, many of the genera found in different abundances between comparison groups were associated with the concentration of plasma trace elements, suggesting another mechanism affecting stone formation. Regarding possible differences between patients with CaOx dehydrate (COD) and monohydrate (COM) lithiasis, Rodriguez et al. compared the gut microbiomes of the above groups [79]. COD lithiasis was characterized by a significant reduction in bacteria number and a significant decrease in the muconutritive *Akkermansia muciniphila* and *Faecalibacterium prausnitzii* compared to COM lithiasis. Both lithiasis patterns were characterized by elements of chronic proinflammatory intestinal dysbiosis, which was more manifest in COD lithiasis. In 2020, Liu et al. focused on the association of gut microbiota with CaOx stone disease through the production of short-chain fatty acids (SCFAs) by the gut microbes [80]. In the study, 153 participants were enrolled (occasional stone formation, recurrent stone formation, and controls). The analysis showed that stone formers exhibited higher microbial diversity than controls. Microbial composition was significantly different in every paired comparison of the participant groups. Several microbes that were found enriched in the control group were associated with the production of SCFAs, which have an anti-inflammatory role in the gut. On the contrary, several microbes that were found to be enriched in stone formers were associated with the triggering of inflammation. In 2021, Zhao et al. performed an analysis focusing on the gut microbiome alterations between controls and stone formers, and, additionally, between patients with different stone compositions [81]. Several differences in the microbe population of stone formers were found, such as the abundance of *Bacteroides* and *Prevotella*, and the depletion of *Lachnoclostridium*, *Blautia*, and *Bifidobacterium*. Moreover, *Prevotella-9* was significantly more abundant in patients with non-calcium-containing stones than in patients with calcium stones, a finding suggesting a role of the microbe in uric acid metabolism. On the contrary, *Pseudobutyrivibrio*, *Lachnoclostridium*, and *Ruminococcus 2* were significantly more abundant in patients with CaOx stones than those with uric acid stones. The researchers suggested that the above pattern of intestinal dysbacteriosis interferes with the mechanism of inflammatory response. According to Xiang et al. [82], gut microbiome alterations can be so suggestive of the presence or absence of stone disease that they can be included in predictive models of the highest accuracy. In the above report, the detection of three microbial genera combined with the values of five clinical parameters increased the predictive accuracy of the tested artificial intelligence (AI)-based models to a maximum AUC of 0.936. The above models referred only to CaOx stone disease. In 2022, Kim et al. enrolled 915 adults in a protocol where the gut microbiomes of controls and incidental and prevalent stone formers were analyzed to uncover possible alterations [83]. All subjects were followed for four years, and the individuals who developed stones during this period were categorized as incidental stone formers, while the individuals diagnosed with urinary stones from the beginning of follow-up were the prevalent stone formers. The gut microbiomes of the groups had the same diversity; however, the prevalent cases presented inequities in the populations of different microbial species. Stone disease was associated with a reduced representation of species evolved in SCFA production, while other metabolic pathways related to respective species perturbations involved the production of uric acid and the citrate cycle. The interaction of diet with gut microbiota as a risk factor for lithogenesis was studied by Yuan et al. [84]. In their report, a group of individuals was divided into four subgroups according to their stone disease status and high- or low-risk dietary pattern. The analysis of the gut microbiomes demonstrated that there was a significantly different abundance between high- and low-risk diets, regardless of the stone disease status. More interestingly, the comparison of stone formers on high-risk diets with stone formers on low-risk diets showed an abundance of microbes relating to lipid metabolism, signaling molecules, and the immune system. In the same way, the comparisons of the other subgroups showed metabolic alterations to a lesser extent. Microbial species that were found to be more abundant in the high- and low-risk diet stone formers were associated with a proinflammatory role, while *Fusicatenibacter* in the low-risk diet/no-stone individuals was correlated with an antioxidant role, which may be protective against lithogenesis. In 2023, a meta-analysis performed by Yuan et al. included eight studies with 356 stone disease patients and 347 controls to pool the existing data on the gut microbiome [85]. The study concluded that patients had a higher abundance of *Bacteroides* and *Escherichia*, while *Prevotella* was abundant in the controls. Significant alterations were found between comparison groups in functional activities relating to oxalate degradation, glycan synthesis, and lipid and carbohydrate metabolism. Overall, the researchers reported that the observed gut dysbiosis may disturb metabolic homeostasis, which acts as a predisposing factor for lithogenesis. In the same year, Zhang et al. processed bioinformatical data, which consisted of 16S rRNA gene sequencing profiles from gut microbiota composition analyses, to find eventual causal associations between gut microbial populations and upper urinary lithiasis [86]. The results demonstrated a protective role for several microbial species (genera *Barnesiella*, *Clostridium sensu_stricto_1*, *Flavonifractor*, *Hungatella*, *Oscillospira*, family *Clostridiaceae1*, class *Deltaproteobacteria*, and order *NB1n*), while the genus *Eubacterium xylanophilus* was found to promote lithogenesis. The majority of the above microbial species had been associated with inflammation-related diseases, which underpins the aspect of stone formation as a manifestation of an aberrant inflammatory immunological response. Interestingly, the researchers found an inverse association (gut microbiota with a propensity to colonize the gut of stone patients) for several microbial strains, which was attributed to the dietary habits and/or the antibiotic usage of the patients. Another study by Wang et al. investigated the gut microbiome differences among urolithiasis patients with and without hypocitruria (HCU) by 16S rRNA gene sequencing [87]. HCU patients had a significantly increased abundance of *Ruminococcaceae_ge* and *Turicibacter* bacterial groups, which have been reported in previous publications as affecting uric acid metabolism. A diagnostic model based on the above bacterial groups was able to distinguish HCU patients with high accuracy.

##### Studies Combining Results from Gastrointestinal and Urinary Tract

During the literature search for articles associating the microbiome with stone disease, we found two reports referring to the combination of analyses of the gut microbiome and the urinary system microbiome. In 2019, Zampini et al. enrolled 67 individuals (among them 24 patients with stones of various chemistry) to analyze the microbial composition and its metabolic characteristics and to uncover the role of microbes in lithogenesis [88]. Regarding the fecal microbes, no significant difference was found in microbial populations, whereas this difference between controls and patients in urine was significant. *Lactobacillus* in the urine of controls and *Enterobacteriaceae* in the urine of patients were the taxa that demonstrated the most apparent differences between the comparison groups. The researchers concluded that the urine microbiome is more relevant for stone formation than the gut microbiome. In 2021, Kachroo et al. pooled the results of six reports to perform a meta-analysis on the association of stone disease with the microbiome in the gut and the urinary tract [89]. Significant differences were found in the microbiome composition not only between patients and controls but also among different age groups, study locations, and stone compositions. *Prevotella* in the gut and *Lactobacillus* in the urinary tract were abundant in healthy individuals, while *Enterobacteriaceae* in both tracts were associated with stone disease. Interestingly, specific strains of *Prevotella* were abundant in the gut of patients, which suggests that the role of *Prevotella* is strain-dependent. Since the meta-analysis included data from the stone microbiome, it was found that *Enterobacteriaceae* were the most abundant species. The researchers concluded that the urinary tract microbiome is more relevant to the mechanism of lithogenesis, which should drive the direction of future studies. In the same year, Suryavanshi et al. pooled the results of six datasets, which contained 16S rRNA gene sequencing data from urine, stone material, and the stool of 201 patients with stone disease and 136 controls, to investigate for eventual associations between microbiome composition and urinary stone status [90]. In contrast to previous reports, the researchers did not remove the rare taxa, which is usually performed to simplify the data, and found that the rare phylotypes provide substantial contributions to the structure of the microbiome. The majority of the species observed in all samples were characterized as rare phylotypes, and the stone material demonstrated the greatest tendency for such phylotypes. Both common and rare phylotypes of urine and stool samples contributed to the differentiation between stone patients and controls. Rare taxa such as *Christensenellaceae*, *Clostridia*, and *Oscillospirales* were significantly enriched in the gut of the controls, while common taxa such as *Bacteroidales* and *Lachnospiraceae*, along with rare taxa such as *Clostridia* and *Bacteroides*, were more abundant in the gut of urinary stone patients. Regarding the urine samples, rare taxa such as *Actinomyces*, *Corynebacterium*, *Sphingomonas*, *Anaerococcus*, and *Bacteroides*, and common taxa such as *Corynebacterium* were found to be enriched in the controls, and common taxa such as *Anaerococcus* and *Corynebacterium* were abundant in the patient group. Recently, Al et al. published the results of their study, which focused on the microbiome perturbations found in the stool, urine, stone material, and, additionally, in the saliva of stone formers [91]. Eighty-three stone formers were recruited along with thirty controls, and their biological material was analyzed by 16S rRNA gene sequencing (for urine and saliva samples), and whole shotgun metagenomic sequencing (for stool samples). Regarding urine samples, *Gardnerella* sp., *Megasphaera* sp., and *Alloscardovia omnicolens* were found to be more abundant in the control group, while *Lactobacillus jensenii* was relatively enriched among stone formers. The respective metabolic processes, which were found to be enriched in stone formers, demonstrated a shift from ‘’healthy” functions (such as vitamin B12 and butyrate biosynthesis) to functions related to virulence factors, antimicrobial resistance elements, and pathobionts. Regarding stool samples, *Oxalobacter formigenes* were not differentially abundant between comparison groups, while stone formers were functionally characterized by the depletion of several essential housekeeping functions (such as protein transport and transcription) and enrichment in virulence and inflammatory processes. The comparison of saliva samples demonstrated only trends but no significant differences.

#### 3.2.2. Urinary Tract Microbiome

##### Studies on the Microbial Composition in Urine

The correlation of microbial perturbations in the urinary tract with stone formation was established many years ago and refers to specific microbial species and specific stone composition. More precisely, the abundance of urease-producing bacteria in the urinary tract has been associated with increased urea metabolism, which produces ammonium and alkalizes urine [10]. The spectrum of urease-splitting bacteria includes several obligate urease-producing bacteria, such as *Proteus* spp., *Providencia rettgeri*, *Morganella morganii*, and several facultative urease-producing bacteria, such as *Enterobacter gergoviae*, *Klebsiella* spp., and *Providencia stuartii* [10]. This specific stone class is characterized as infection stones and encompasses minerals such as struvite, carbonate apatite, and ammonium urate. In recent years, scientific data suggest that infection stones and other stone compositions may be associated with a greater variety of microbes than the already known urease-producing bacteria (Table 1). Indeed, a report by Flannigan et al. described the results of the urine analysis of a patient group who underwent surgery for their homogenous struvite stones or mixed struvite stones with calcium phosphate, CaOx, calcium carbonate, and uric acid [92]. The most common isolated microbe was *Proteus*; however, *Escherichia Coli* and *Enterococcus* were also found frequently. Interestingly, classical urease-producing bacteria were isolated in only 30% of the patients. Moreover, no significant differences were found between the cases with homogenous struvite and the cases with mixed composition. The study suggested that non-urease-producing bacteria may coexist with the respective urease-producing bacteria, and in case the urine sample is over-populated by the former, the latter cannot be isolated. In 2017, Amimanan et al. studied the possible role of *Escherichia Coli* in CaOx stone formation by comparing microbe strains from stone formers with respective strains from patients with *Escherichia Coli* infection and no urinary stones [93]. Proteomic analysis of both strain groups showed that a protein known as elongation factor Tu (EF-Tu) was the most abundant parameter characterizing the strains of stone formers. The lithogenic effect of EF-Tu was confirmed experimentally by crystallization assay and measurement of crystal aggregation after neutralization with anti-EF-Tu antibody. More recently, molecular testing through 16S rRNA sequencing has demonstrated the presence of microbial dysbiosis also in the urinary tract of stone formers. According to a study by Xie et al. [94], the comparison of the urine from healthy controls with respective samples of matched CaOx stone formers revealed a reduced species diversity and microbial composition in the patient group. Moreover, the urine from the kidney pelvis of patients was almost similar to the urine from the bladder of the same individuals, and only two strains (*Anoxybacillus* in kidney pelvis, *Fusobacterium* in bladder) showed significant differences. The most characteristic microbial taxa were *Prevotella* in the bladder of controls and *Acinetobacter* in the kidney pelvis of patients. According to data from other pathologic conditions, the microbial species, which were found to be enriched in the above patients, are considered proinflammatory, and the respective enriched metabolic pathways were related to ion channels. In 2020, Liu et al. investigated the further evolvement of the urinary tract microbiome in patients with kidney stones under the gradual manifestation of arterial hypertension, which demonstrates a disproportionally high incidence among stone formers [95]. The analysis showed significant differences between healthy controls and normotensive and hypertensive kidney stone patients in terms of the bacterial composition in their urine. Moreover, the urine microbiome of hypertensive patients exhibited higher diversity, and its most abundant microbe was *Sphingomonas*, while in normotensive patients and healthy controls, they were *Staphylococcus* and *Gardnerella,* respectively. The researchers concluded that further alterations of the urinary tract microbiome occur simultaneously with the increasing kidney damage from the stone burden and the development of arterial hypertension. Another study by Goloshchapov et al. demonstrated that not only the qualitative alterations in terms of microbial diversity and composition but also the total microbial burden play significant roles in the increase in the recurrence rate in stone formers [96]. The above protocol included 163 patients with a high tendency for stone relapse, whose urine samples had an extremely high level of bacterial burden, and 35 healthy individuals with much lower bacterial counts. Tamm–Horsfall protein (THP) represents the primary stabilizer of the colloidal properties in urine and has a protective role against lithogenesis. In the patient group, THP complexes had a significantly larger size than in healthy controls. The researchers considered this enlargement as a structural failure of THP, which accompanies the increased bacterial counts of the patients. Interestingly, 12 patients underwent metaphylaxis therapy, which dramatically reduced the bacterial counts and the size of THP complexes to near the normal range, suggesting an inverse correlation of bacterial burden to the stability of the urine colloidal properties. In 2021, Shen et al. analyzed the microbiome in urine samples of patients with CaOx stones and found the existence of two main clusters of respective microbes (OA1, OA2), which had clinical implications, since the urine of OA1 patients had a significantly higher white blood cell per high-power field count, suggesting the triggering of a more intense inflammatory reaction [97]. Indeed, OA1 was characterized by a relative abundance of *Enterobacteriaceae,* at the level of 80.1%, while the microbial population of OA2 was significantly more diverse. *Enterobacteriaceae* had significantly positive associations with pathways relating to infection, such as nitrogen metabolism and bacterial invasion of epithelial cells, while the relatively abundant *Bifidobacterium* and *Lactobacillus* in the OA2 cluster are considered probiotics. An innovative methodology of urine sampling was applied by Yang et al. to uncover the microbial strains with lithogenic effect and included the analysis of urine from both sides of patients with unilateral CaOx stones [98]. The microbiome comparison of the healthy side to the stone-bearing side revealed 26 significantly different bacteria, and *Enterobacter cloacae* demonstrated the widest difference. Bioinformatics results suggested that ion binding and signal transduction are the pathways that correlate *Enterobacter cloacae* with CaOx lithogenesis. The study included in vivo experiments, which confirmed the pro-lithogenic role of *Enterobacter cloacae* in environments with preexisting metabolic disorders. In another study by Kachroo et al. [99], urine samples of a patient group with pure CaOx stones were analyzed and compared to respective samples of healthy controls in terms of microbial functions across prokaryotic, fungal, viral, and protozoan domains. The analysis showed that non-prokaryotic domains did not differentiate between patients and controls, while *Lactobacillus* was the most significant differentiating factor, demonstrating a significant enrichment in the control group. The urine microbiome of the patient group showed reduced levels of genes associated with oxalate metabolism, proteolysis, and transmembrane transport. In 2022, Gao et al. performed a multi-omics study to investigate differences in urinary microbiomes and the respective metabolic alterations in patients with kidney stones [100]. Significant variation was found in terms of microbial diversity and composition depending on disease status (patients, controls) and gender, which may be associated with the higher incidence of stone disease in males. Metabolomic analysis showed that four metabolites, which were associated with metabolic acidosis, nicotine metabolism, anti-inflammation, and lipid metabolism, showed significant differentiation between patients and controls. Hong et al. applied a novel microbial sequencing technology (2bRAD-M) to uncover the microbiome differences between the stone-bearing kidney pelvis and the healthy kidney pelvis of a patient group with unilateral stone disease of various chemical compositions [101]. The analysis showed that the overall microbial composition of the comparison groups was similar. At the species level, four microbial strains (*Previotella bivia*, *Lactobacillus iners*, *Corynebacterium aurimuntum*, *Pseudomonas sp_286*) were enriched in the healthy kidney pelvis of the patients. The above study was the first performed with the novel sequencing method, which allowed the characterization of microbiomes at the species resolution.

##### Studies on the Microbial Composition in Stone Material

Stone formation takes place in the urinary tract under the effect of the urine microbiome, which renders plausible the hypothesis of uncovering microbiome elements in stone material. Indeed, several studies have reported on microbial strains isolated from stone material with a role in lithogenesis, and among the recent publications, we selected two reports on the above topic. In 2017, Ansari et al. studied the eventual role of nanobacteria, which were isolated from stone samples, in stone formation [102]. Nanobacteria represent nano-scale structures, which are considered ultra-small bacteria with unknown properties by several researchers, while other specialists have characterized them as structures of abiotic nature. Nanobacteria, which commonly carry mineralized shells made of carbonate apatite, were isolated from all patients of the study with apatite kidney stones, but not from patients with other chemical compositions. Based on the above results, the researchers suggested that nanobacteria may play a role in the initiation of lithogenesis through their biomineralization activity. Similarly, Saw et al. performed a combined microscopy and DNA sequencing analysis on the stone material of a patient group, which included 18 CaOx stone formers, one case with brushite and another with struvite stones [103]. Bulk-entombed DNA was isolated and sequenced from 11 of the 18 CaOx stones and both brushite and struvite stones. The analysis demonstrated the presence of a low-diversity bacterial population (*Actinobacteria*, *Bacteroidetes*, *Firmicutes*, *Proteobacteria*) and the simultaneous presence of fungal strains (*Aspergillus* niger). Microscopy was performed on five CaOx cases, without evidence of microbes, while the respective analyses on brushite and struvite cases exhibited entombed bacteria in the amorphous hydroxyapatite mass.

##### Studies Combining Analysis in Urine and Stone Material

The combination of analyses of stone and urine samples carries the advantage of providing the most comprehensive data on the urinary tract microbiome in urinary stone patients. During our literature search, we found four reports with the above methodology, and we summarized the respective results. In 2017, Parkhomenko et al. published the microbial analysis results of samples of 50 struvite stone patients, who were included in the protocol from an initial cohort of 1191 stone patients [104]. Stone cultures were positive in 72% of the struvite stone patients, while the respective percentage in preoperative urine samples was 48%. Classical urea-splitting bacteria were isolated only in half of positive stone cultures, and two-thirds of patients with negative stone cultures had a history of positive urine culture for urea-splitting bacteria in the last 12 months before surgery. The discordance rate between stone and urine cultures reached the level of 46%. The researchers suggested that after a period of stone formation by an initial inciting microorganism, secondary colonization by non-urease-producing microbes explains the isolation of respective strains, such as *Enterococcus*. Moreover, the study demonstrated that preoperative urine cultures do not accurately predict the microbial strains within the stone material, which has practical implications for patient prophylaxis against infectious complications. In 2020, Dornbier et al. performed a microbial analysis of urine and stone samples of 52 patients with mainly calcium-based stones [105]. The researchers conducted 16S rRNA gene sequencing and cultures with enhanced technique and found that 29 stones were sequence positive, while negative stone samples were mostly of CaOx composition. Among sequence positives, 12 stones contained 1–2 dominant microbial taxa, which were found to be enriched in terms of relative abundance compared to the urine samples. Extended-technique cultures were positive in nine of the above twelve stone samples, which confirmed the feasibility of the isolation of enriched bacteria through culture methods. Another study by Dornbier et al. examined the association of the urinary tract microbiome with the diagnosis of metabolic syndrome in urinary stone patients [106]. The extended culture technique showed that bacteria were found more frequently and in higher abundance among stone patients with metabolic syndrome. 16S rRNA gene sequencing validated the above results and demonstrated that particular microbial genera were significantly more enriched in the stone material of several patients than in their respective urine samples. The researchers suggested that microbes that were found to be enriched in stone samples compared to urine were associated with the process of stone formation. In 2023, Lemberger et al. analyzed the microbial population in samples (urine + stone material) of 100 consecutive patients and healthy controls (urine), aiming to uncover significant characteristics of the microbiomes of the patient group [107]. The analysis was performed via 16S rRNA gene sequencing, which detected bacteria in 24% of stone samples. No uric acid stone displayed any microbial strain, while the highest positive signals were detected in apatite and apatite/weddellite/whewellite stones. The patient group with identifiable microbial strains in their respective stone samples displayed a longer length of stay and a higher rate of postoperative complications after percutaneous nephrolithotomy. Interestingly, patients with features of metabolic syndrome had stones colonized with classical gastrointestinal microbial strains (*Enterococcaceae*, *Enterobacteriaceae*), while metabolically fit patients had stones with enrichment of *Ureaplasma* and *Staphylococcaceae*. The researchers concluded that metabolic syndrome in stone patients is associated with a distinct stone microbiome.

#### 3.2.3. The Role of Pharmaceutical Agents Acting on the Microbiome in Stone Formation Risk

##### Studies Referring to *Oxalobacter formigenes* Supplementation and Probiotics

Since *Oxalobacter formigenes* depends on oxalate degradation as an energy source and many studies reported an inverse correlation of *Oxalobacter formigenes* colonization in the gut with stone formation risk, many researchers hypothesized that increasing the microbe count in the gut by supplementation would confer a reduction in lithogenesis. In 2017, Hoppe et al. reported the results of a randomized, placebo-controlled study, which aimed at the assessment of the effect of *Oxalobacter formigenes* administration (OC-5) on 28 patients with PH [108]. After eight weeks of treatment, the main endpoints (urinary oxalate excretion, plasma oxalate concentration) showed no significant difference, while the microbe cell count was significantly increased in treated patients. The treatment was well tolerated. Another *Oxalobacter formigenes* supplementation form (OC-3) was evaluated for 24 weeks in a randomized, placebo-controlled study by Milliner et al. [109]. In the respective protocol, 36 patients with a diagnosis of PH were randomized into comparison groups. Among the main endpoints, urinary oxalate excretion, responder number, and stone events were not different between treatment and placebo, while there was a trend toward increased total plasma oxalate in the placebo group, which suggests that OC-3 may increase the oxalate transfer plasma to the gut. Nevertheless, the above comparison did not show a significant difference, which may be attributed to the short duration of exposure to the OC-3 agent. According to a systematic review by Lieske et al., the presence of *Oxalobacter formigenes* in the human gut correlates to the stone status of the individuals, but neither the oral administration of *Oxalobacter formigenes* nor the trials of Lactobacillus-containing probiotics demonstrated a significant and sustained protective effect against lithogenesis in the urine chemistry of the study participants [110]. In 2021, Tavasoli studied the effect of a probiotic supplement that contained Lactobacillus acidophilus and Bifidobacterium animalis lactis on the 24-h excretion of urine oxalate among recurrent calcium stone formers [111]. According to the in vitro part of the study, only Lactobacillus acidophilus demonstrated a significant oxalate-degrading activity. Moreover, the patients who were randomized into the probiotic group showed no differences in terms of urine oxalate excretion compared with the patients of the placebo group.

##### Studies Referring to the Association of Microbiome Perturbation After Antibiotic Exposure and Stone Formation Risk

Antibiotics are recognized as major disruptors of the intestinal microbial community, which in turn can lead to several acute or chronic pathologic conditions, such as antibiotic-associated diarrhea, and recurrent *Clostridium difficile* infection. Since there are many reports associating gut microbiome imbalance with lithogenesis, it is plausible to hypothesize that antibiotic exposure may increase stone formation risk. In 2017, Tasian et al. conducted a population-based, case-control study, in which the comparison groups were matched in terms of major confounders [112]. Among twelve classes of oral antibiotics, exposure to any of five antibiotic classes (sulfonamides, cephalosporines, fluoroquinolones, nitrofurantoin/methenamine, penicillin) in the period of 3–12 months before stone disease diagnosis was associated with nephrolithiasis. These associations remained significant after adjustment for confounding parameters, and their magnitude was higher for younger patients and exposure during the period of 3–6 months before the stone diagnosis. Interestingly, these associations (except penicillin) remained significant 3–5 years after exposure. In 2019, Ferraro et al. reported the results of a prospective cohort study, which included 5010 women of three age ranges (40–49, 40–59, 20–39) and aimed to find possible associations of stone disease with antibiotic exposure [113]. The researchers stated that the cumulative antibiotic administration for a time period > 2 months was associated with significantly higher stone formation risk across all age ranges, and the above associations remained after adjustment for potential confounders. More recently, Nazzal et al. studied the effect of antibiotic exposure on *Oxalobacter* colonization in the gut [114]. The researchers recruited 65 healthy subjects, which included 19 individuals planned for *Helicobacter pylori* eradication through antibiotic therapy and 46 controls. Serial analyses of fecal samples demonstrated significant long-lasting suppression of *Oxalobacter* in antibiotic-treated subjects, while the control group retained a stable *Oxalobacter* population and a higher diversity of microbial strains in the gut. Regarding the urine chemistry, urinary pH was increased after antibiotics, but urinary oxalate was similar between comparison groups. Similarly, Thongprayoon et al. reported in their publication that antibiotic use does not increase the risk for urinary stone disease [115]. In their study, they recruited 1247 symptomatic stone formers and 4024 matched controls, and in their analysis, no increased risk of symptomatic stone disease was found after antibiotic use and after adjustment for comorbidities and exclusion of participants with prior urinary symptoms. According to the researchers, the increased risk for symptomatic stone disease that was reported previously may be attributed to antibiotic administration for urinary symptoms, which are triggered by under-recognized kidney stones (reverse causality).

## 4. Discussion

According to the included data on the effect of lifestyle elements and the microbiome, both factors are associated with lithogenesis/stone recurrence, yet the applicability of these findings lies at different maturation degrees since the research on the microbiome effect is significantly more recent. Both factors have metabolic implications for the individuals, and their manipulation can contribute to the prevention of urinary stones, which comprise a disease considered a chronic metabolic condition [116]. Since the public health burden of urolithiasis demonstrates an increasing trend, the application of the above factors in health strategies for primary/secondary prevention is necessary [116].

**Figure 3:**
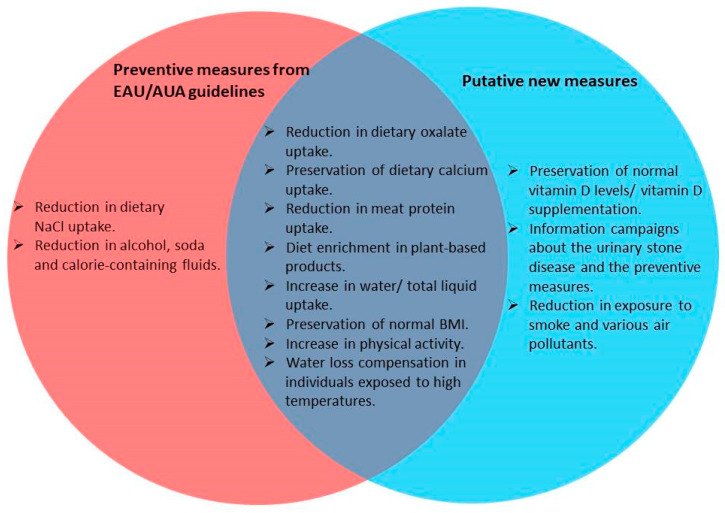
Recommendations overlap between urological guidelines and included studies.

Regarding the effect of lifestyle factors on lithogenesis, numerous publications have reported on this association, and the respective evidence is adequately strong such that preventive measures that are based on the manipulation of lifestyle conditions are already included in urolithiasis guidelines [10,11]. Among the included reports in the current review, we found that the majority of interventions/parameters under investigation have already been interpreted into preventive strategies in the above guidelines, while additional parameters with a putative role in urolithiasis are the maintenance of normal vitamin D levels/supplementation, the awareness degree of the patients about the mechanisms of lithogenesis/the role of stone prevention, and the exposure to various air pollutants in the occupational or other environments (Figure 3). Vitamin D levels seem to comprise a cornerstone in calcium metabolism, affecting the amount of excreted calcium by urine and the risk for stone formation. Additionally, several studies demonstrated a correlation between socioeducational factors/behavioral patterns and urolithiasis prevalence. The educational level, which is also affected by socioeconomic status, may affect the understanding of the lithogenesis process and the adherence to preventive measures. Consequently, a campaign to inform the public may be considered as an additional strategy to reduce the impact of stone disease on the general population. Among the proposed lifestyle interventions, dietary modifications represent the main prevention bundle against lithogenesis, yet these modifications should be further communicated to medical professionals and the affected individuals to ensure a significant effect in terms of lithogenesis risk reduction (Table 2).

Relating to the evidence strength of the included reports on the effect of lifestyle factors, the majority of the studies were longitudinal and presented their results after adjustment for eventual confounders. Moreover, about half of the included studies had a prospective design. Most of the proposed methods are already included in the preventive strategies of various urological societies, yet the results of applying such lifestyle measures are not satisfying since the prevalence of stone disease has nearly doubled over the last few years [116]. According to Rodgers et al., the increasing incidence and prevalence of urolithiasis in the last 50 years reflects the fact that lifestyle adaptations and pharmacotherapy have no major effect on the lithogenesis risk [117]. Furthermore, the authors state that the respective guidelines on urolithiasis prevention only reformulate the existing knowledge without offering any breakthrough strategy, whereas multi-omics studies can provide new perspectives to apply effective urolithiasis prevention.

**Figure 4:**
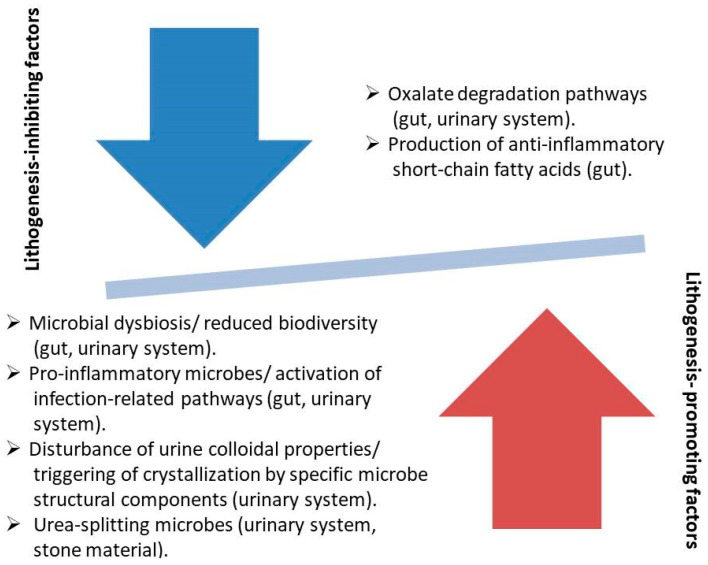
Microbiome-dependent mechanisms with an inhibiting/promoting role in urinary stone formation.

Regarding the association of the microbiome with lithogenesis, the available publications are more recent and numerically limited, while their methods and results are mostly heterogeneous. Relating to the reports published from 2015 to the present, several putative microbiome-dependent mechanisms seem to affect the risk of stone disease. These mechanisms include the oxalate-degrading activity and the production of anti-inflammatory SCFAs by the microbes as lithogenesis-inhibiting factors (Figure 4). On the contrary, the activation of infection-related pathways, the alterations of urine colloidal properties, and the pH increase by urea-splitting microbes, which are accompanied by alterations in the relative population composition/activity of different microbial species, seem to play a lithogenesis-promoting role (Figure 4). Contrary to the studies related to lifestyle factors, the majority of the included microbiome studies were cross-sectional and presented non-adjusted results for confounders, which reduces the strength of the respective evidence. Furthermore, almost all reports used culture-independent methods to investigate the microbial populations/functions (16S rRNA gene sequencing, shotgun metagenomics sequencing).

The disturbance of intestinal microbial composition, which is defined with the term intestinal dysbiosis, seems to be associated not only with urolithiasis but also with other renal disorders, such as chronic kidney disease, the status of end-stage renal disease hemodialysis, and immunoglobulin A nephropathy [118]. The above findings are based on high-technology culture-independent techniques, and suggest the presence of a gut–kidney axis, which may be decisive for the maintenance of healthy status or the development of several renal diseases. Regarding the perspective of microbiome manipulation as a therapeutic measure against health conditions, several methods have been proposed, such as fecal microbiota transplantation (FMT), the application of dietary adaptations, and the administration of isolated/engineered microbes or their respective proteins/metabolites, but only a limited number of these methods have been approved by the official health authorities [119]. Among the above methods, FMT represents the most studied microbiome manipulation approach, and there are already promising results from its application in the management of gastrointestinal tract diseases. More precisely, several reports support FMT as an effective option for the management of pancreatic conditions, such as in patients with acute pancreatitis, or type 1 diabetes [120]. Similarly, several publications report the positive role of FMT in patients with gastroenterological and neurological cancers or other tumor forms [121]. Regarding the effect of FMT on lithogenesis, our search revealed only reports from animal models. In the majority of these studies, the researchers performed FMT on rodents, which in turn underwent urine chemistry alterations with subsequent increased/decreased lithogenesis risk (depending on the fecal microbiome donor) [122,123].

**Figure 5:**
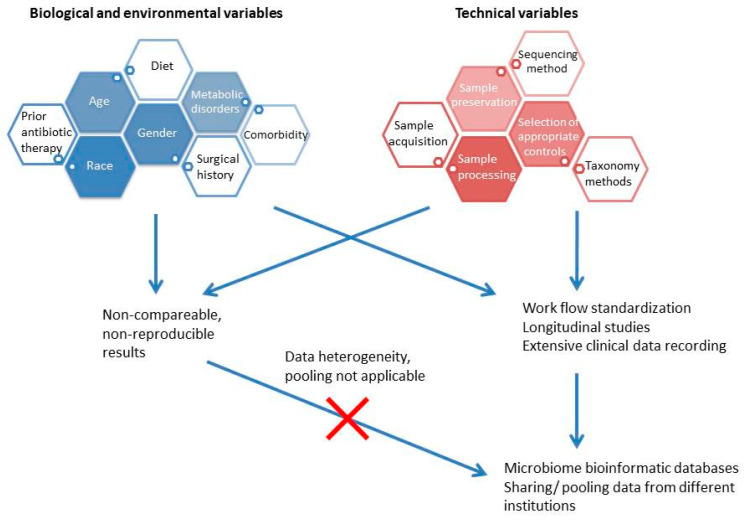
Systematic factors of bias in microbiome studies.

Microbiome research may offer innovative methods to understand and prevent urolithiasis, but currently, it is accompanied by several shortcomings, which impact its applicability. The most important issue is the lack of standardization, which leads to heterogeneity in the sample material, method of sample acquisition, and preservation [124]. Moreover, the application of various sequencing technologies leads to different processing protocols, which has a direct effect on the results of the analyses [125]. The above technical issues in microbiome research (Figure 5) can be solved by scientific consensus on the application of standardization rules. Such initiatives have already taken place relating to microbiome studies for urolithiasis, and they are expected to contribute to the generation of standardized protocols, which will allow the production of comparable results and the pooling of these data into bioinformatics databases [126].

Additionally to the technical variables, several biological/environmental factors have to be recorded and taken into account in the context of performing microbiome research, since they directly affect the microbial populations/functions (Figure 5). These factors include the patient’s characteristics (age, sex, race), and the medical history of the patient (surgeries in the past, metabolic disorders, recent antibiotic therapy) [127]. The registration of the above data is expected to allow the exclusion of confounding effects and the production of objective results, which can be pooled and further analyzed. An essential issue relating to the detection of microbiome perturbations is the putative existence of a core microbiome, which may be used as a reference for comparisons with the analyses of various diseases. To the present, there is no consensus as to whether a core microbiome exists and at which level (different microbial strains, microbial functions, other) it could be defined [128]. Additionally, the core microbiome will possibly include viruses and fungi, which have been investigated only to a very limited extent. Both the technical variables of microbiome analysis and the biological factors that shape the microbiome characteristics inflict substantial challenges in the process of integrating microbiome-based methods in urolithiasis prevention.

## 5. Conclusions

The included data of the current review suggest that a highly effective stone prevention strategy and an inversion of the increasing trend in urolithiasis incidence/prevalence are not likely for the near future. Regarding lifestyle-based prevention measures, the recent literature mostly repeatedly proposes the methods that are already included in urological guidelines and bring only a mild benefit to stone formers. Microbiome research comprises one of the promising scientific sectors in the context of urolithiasis prevention. However, the microbiome is subject to influence from numerous clinical/biological factors, and the respective analysis depends on a variety of technical parameters/procedural specifications. The standardization of the analysis procedures and data pooling from well-designed studies comprise the only way to achieve unbiased results that will be applicable in the clinical practice of urinary stone prevention.

## Figures and Tables

**Table 1:** Gut/urinary microbiome associated with urolithiasis.

	Microbes Acting as Lithogenesis Inhibitors	Microbes Acting as Lithogenesis Promoters
Gut	*Prevotella* (species) *Ruminococcus* (genus) *Lactobacillus* (genus) *Faecalibacterium* (genus) *Bifidobacterium* (genus) *Oscillospira* (genus) *Akkermansia* (genus) *Lachnoclostridium* (genus)	*Bacteroidetes* (phylum)
Urinary system	*Lactobacillus* (genus) *Gardnerella* (genus) *Corynebacterium* (genus) *Prevotella* (species)	*Enterobacteriaceae* (family) Urea-splitting microbes

**Table 2:** Overview of dietary recommendations for general prevention of urolithiasis and further research.

Standard Recommendations	Protective Effect on Urine Chemistry	Quantitative Limit ^a^
Reduction in dietary NaCl uptake	Decreased calcium concentration in urine Increased citric acid concentration in urine	≤4–5 g/day
Reduction in sodas and calorie-rich beverages	Increased urinary pH Decreased calcium concentration in urine	Not defined
Reduction in dietary oxalate uptake	Decreased oxalate concentration in urine	Not defined
Reduction in non-dairy animal protein uptake	Increased urinary pH Decreased uric acid concentration in urine	≤0.8–1.0 g/kg/day
Preservation of dietary calcium uptake	Decreased oxalate concentration in urine	1–1.2 g/day
Increase in vegetable and fiber uptake	Increased urinary pH Increased citric acid concentration in urine	Not defined
Increase in fluid uptake (preferred fluid: water, increased fluid compensation under exposure to high temperatures)	Dilution of lithogenic components	Fluid volume: 2.5–3 L/day (diuresis volume: 2.0–2.5 L/day)
**Dietary interventions for further investigation**	**Protective effect on urine chemistry**	
Vitamin D supplementation in individuals with vitamin D deficiency	Unclear	
Tea consumption	Unclear	

^a^ According to the European Association of Urology and the American Urological Association.
